# Field Exploitation of Multiple Functions of Beneficial Microorganisms for Plant Nutrition and Protection: Real Possibility or Just a Hope?

**DOI:** 10.3389/fmicb.2020.01904

**Published:** 2020-08-05

**Authors:** Jolanta Kowalska, Józef Tyburski, Kinga Matysiak, Bartosz Tylkowski, Eligio Malusá

**Affiliations:** ^1^Institute of Plant Protection, Poznań, Poland; ^2^Department of Agroecosystems, University of Warmia and Mazury in Olsztyn, Olsztyn, Poland; ^3^Eurecat, Centre Tecnològic de Catalunya, Tarragona, Spain; ^4^Research Institute of Horticulture, Skierniewice, Poland

**Keywords:** biofertilizers, biopesticides, microbial consortia, multifunctional bioproducts, plant growth-promoting microorganisms, endophytes

## Abstract

Bioproducts, i.e., microbial based pesticides or fertilizers (biopesticides and biofertilizers), should be expected to play an ever-increasing role and application in agricultural practices world-wide in the effort to implement policies concerned with sustainable agriculture. However, several microbial strains have proven the capacity to augment plant productivity by enhancing crop nutrition and functioning as biopesticides, or vice-versa. This multifunctionality is an issue that is still not included as a concept and possibility in any legal provision regarding the placing on the market of bioproducts, and indicates difficulties in clearly classifying the purpose of their suitability. In this review, we overview the current understanding of the mechanisms in plant-microbe interactions underlining the dual function of microbial strains toward plant nutrition and protection. The prospects of market development for multifunctional bioproducts are then considered in view of the current regulatory approach in the European Union, in an effort that wants to stimulate a wider adoption of the new knowledge on the role played by microorganisms in crop production.

## Introduction

Chemical, synthetic plant protection products (PPPs) and mineral fertilizers are criticized mainly for their potentially negative effects on human health (Bennekou, 2019) and/or the environment (Norse, 2005; Huang et al., 2017) including non-renewable resources depletion (Chojnacka et al., 2020), and overall negative impact on biodiversity (Mozumder and Berrens, 2007; Sánchez-Bayo and Wyckhuys, 2019). Because of these concerns, the application of sustainable crop production methods is required by consumers as well as by legal provisions (Marrone, 2019).

In this context, although microbial-based pesticides or fertilizers (hereafter biopesticides and biofertilizers, respectively) would not be expected to fully replace chemical pesticides and mineral fertilizers, they could play an ever-increasing role and application in agricultural practices world-wide (Alabouvette et al., 2012; Kurek and Ozimek, 2013).

The multifunctional use of bioinocula represents an issue that is emerging from several researches (Harman, 2011; Lopes et al., 2018), and could further support the development, marketing and application of microbial-based products. Yet, this prospective is neither included as a concept nor as a possibility in any legal provision dealing with the marketing of bioproducts, while it has started to be appraised in bioproducts for human consumption (Ma et al., 2019).

In this review we are summarizing recent findings on the multiple effects of microorganisms suitable as biofertilizers or biopesticides, in light of the intricated interactions between plants and microorganisms, in an effort to foster the discussion on new products that could find a better acceptance by farmers because of their multifunctional properties.

## Plant-Microbial Inocula Interactions as the Basis for Multifunctional Bioproducts

The interaction between plants and beneficial fungi involves elicitors released by them which include several metabolites, including volatiles (Shoresh et al., 2010; Morath et al., 2012). These compounds function as signal transduction in plants, and as a result both the plant proteome and transcriptome are affected, as it has been observed with *Trichoderma* (Marra et al., 2006; Shoresh and Harman, 2008; Lorito et al., 2010; Lombardi et al., 2020) or arbuscular mycorrhizal fungi (Jung et al., 2012; Cameron et al., 2013; Rivero et al., 2015; Adolfsson et al., 2017). The effect of these modifications is translated into increased plant growth, particularly under stress, improved nutrient use efficiency, acquisition of a systemic resistance to diseases that goes beyond the commonly induced systemic and acquired resistances (Shoresh et al., 2010; Cameron et al., 2013). Qualitatively similar effects are induced in plants by rhizobacteria: the interactions involve different chemical compounds (Abriouel et al., 2011; Fickers, 2012; Lopes et al., 2015; Jasim et al., 2016) as well as priming (Brencic and Winans, 2005; van Wees et al., 2008). Mechanisms include induction of the plant innate immune response system (Jain et al., 2011) or acquired systemic resistance (Iavicoli et al., 2003; Choudhary and Johri, 2009), alteration of plant functional traits (Friesen et al., 2011) and prevention of pathogen settling (Bakker et al., 2012).

On the other hand, growth promotion in bacteria derives mainly from the synthesis of several plant growth hormones (Arkhipova et al., 2005; Xie et al., 2014; Radhakrishnan and Lee, 2016) or their indirect regulation through production of volatile organic compounds (Tahir et al., 2017; Rath et al., 2018) and 1-aminocyclopropane-1-carboxylate deaminase (Glick et al., 2007), as well as the solubilization or mineralization of mineral nutrients (Malusá et al., 2016). A key role in interaction between plants and microorganisms seems to be played by pattern recognition receptors (PRRs), localized in the plants’ plasma-membrane, which allow to recognize beneficial microbe/pathogen-associated molecular patterns (Boller and Felix, 2009; Zipfel, 2014; Trdá et al., 2015).

Nevertheless, the relation between plants and beneficial microorganisms inocula occurs within a wider framework of interactions, including those with the plant microbiome (Berg et al., 2017; Fadiji and Babalola, 2020) as well as with the soil physical, chemical and biological characteristics (Bardi and Malusá, 2012; Vimal et al., 2017), which all contribute to increase the complexity in developing sustainable management practices and agricultural products such as biofertilizers and biopesticides as well as for better exploiting their characteristics.

## Biopesticides and Plant Growth Promotion

Several biopesticides have been developed to protect plants from pests since the mid-twentieth century (Copping and Menn, 2000; de Faria and Wraight, 2007) and among them several entomopathogenic fungi (e.g., Beauveria spp., Zimmermann, 2007) and bacteria (e.g., *Bacillus thuringiensis*, de Almeida Melo et al., 2016) are currently used in crop protection. However, recently published studies have provided evidence for the involvement of entomo- or myco-pathogenic microorganisms in promoting plant growth, thus opening new opportunities of their multifunctional use (Vega et al., 2009; Lacey et al., 2015; Table 1). Examples with entomopathogenic fungi include the significant increase in onion yields after *Metarhizium anisopliae* sprays (Maniania et al., 2003) or in growth of soybean seedlings (Khan et al., 2012) or maize plants (Liao et al., 2014) or cotton (Lopez and Sword, 2015) after soil inoculation with different entomopathogenic species. The mechanism of growth promotion is related to the transfer of nitrogen, also from the parasitized pest, which occurred in both leguminous and gramineous species (Behie et al., 2012). However, production of siderophores (Jirakkakul et al., 2015) or increased uptake of iron (Sánchez-Rodríguez et al., 2015) have also been demonstrated to occur in plants colonized with the entomopathogenic *B. bassiana*. The production of the auxin indole-3-acetic acid was likewise found to be associated to several *Metarhizium* and *Beauveria* strains (Liao et al., 2017). Nevertheless, as for the expression of the full efficacy in insect pests’ control, the ability of fungal entomopathogens to promote plant growth has resulted to depend on the inoculation method (Jaber and Enkerli, 2016, 2017) or the inoculation rate (García et al., 2011).

**TABLE 1:** Microbial strains showing plant protection and growth promotion effects.

**Strains**	**Effect**	**References**
Trichoderma atroviridae	Plant growth promoter Biocontrol of fungal pathogens	Marra et al., 2006
Trichoderma harzianum	Solubilization of phosphates Biocontrol of Fusarium disease	Altomare et al., 1999; Martínez-Medina et al., 2009
Trichoderma viridae	Plant growth stimulator Modulate rhizosphere microbial and improve N uptake	Fiorentino et al., 2018; Znajewska et al., 2018
Trichoderma spp.	Activator of plant physiological processes Elicitor of plant resistance system and plant growth stimulator	Kowalska et al., 2012; Lombardi et al., 2020
Glomus mosseae and Rhizobium leguminosarum	Biocontrol Fusarium root rot	Dar et al., 1997
*Bacillus amyloliquefaciens*	Biocontrol of root pathogens Induce systemic resistance, protect plants against attacks of pathogenic microbes, viruses, and nematodes	Chowdhury et al., 2015; Borriss, 2020
*Bacillus subtilis*	Plant growth	Arkhipova et al., 2005
*Bacillus mojavensis*	Plant growth modulators	Rath et al., 2018
*Bacillus methylotrophicus*	Supports plant growth and enhances nutritional metabolites	Radhakrishnan and Lee, 2016
*Metarhizium anisopliae*	Plant growth and mitigates salt stress Biocontrol of Trips tabaci	Maniania et al., 2003; García et al., 2011; Khan et al., 2012; Lopez and Sword, 2015
Clavicipitaceous endophytes	Suppression of the plant diseases	Kuldau and Bacon, 2008
*Metarhizium robertsii*	Promotes root growth Antagonism to Fusarium solani	Sasan and Bidochka, 2013; Liao et al., 2017
*Beauveria bassiana* and *Metarhizium*	Reduce fungal and virus disease	Jaber and Salem, 2014
*Beauveria bassiana* and *Metarhizium brunneum*	Insect pests’ control and promote plant growth	Lopes et al., 2015; Jaber and Enkerli, 2016
*B. bassiana*	Alleviates Fe chlorosis Biocontrol of downy mildew	Jaber, 2015; Sánchez-Rodríguez et al., 2015
*Pseudomonas* spp.	Plant growth-promoting Mobilization of insoluble forms of K Biocontrol of Phytophthora infestans	Santoyo et al., 2012; Meena et al., 2014; De Vrieze et al., 2018
*Pseudomonas* fluorescens	Induces systemic resistance	Iavicoli et al., 2003
Microbial consortia	Biocontrol of Sclerotinia sclerotiorum	Jain et al., 2011
Rhizobacteria	Priming, induction of the plant immune response system prevention of pathogen settling Pathogen suppression	van Wees et al., 2008; Jain et al., 2011; Bakker et al., 2012; Islam et al., 2019
*B. bassiana*, *Lecanicillium dimorphum*	Modulates plant defense responses and energy metabolism	Gómez-Vidal et al., 2009
*Phanerochaete chrysosporium*	P-solubilizing filamentous fungi against Fusarium wilt	Khan and Khan, 2001
*Paenibacillus kribbensis*	Potassium and phosphate-solubilizing capacity and reduce of several cotton and wheat soil-borne pathogens	Zhang et al., 2013

Increased plant growth mediated by entomopathogenic fungi could result from the suppression of the plant diseases (Kuldau and Bacon, 2008; Jaber, 2015) or from a combination of reduced disease severity and more vigorous development of the plants as observed with *Beauveria* and *Metarhizium* strains and fungal or virus pathogens (Sasan and Bidochka, 2013; Jaber and Salem, 2014). In these cases, the mechanisms could derive from the capacity of entomopathogenic fungi to elicit the expression of photosynthesis- and energy metabolism-related proteins as well as plant defense responses (Gómez-Vidal et al., 2009).

Among pathogen biocontrol fungi, the dual effect of Trichoderma application has been observed in several studies. *T. harzianum* T-22 proved to solubilize *in vitro* insoluble rock phosphate likely by both chelation and reduction processes, since no release of organic acids nor acidification were observed (Altomare et al., 1999). Trichoderma-based products were shown to modulated rhizosphere microbial populations, improving nutrient uptake efficiency, yield, and nutritional quality of leafy vegetables (Fiorentino et al., 2018) or of strawberry plants (Lombardi et al., 2020). Dipping roots of strawberry cuttings in a suspension of *T. asperellum* prior to planting followed by foliage applications during the vegetation season stimulated plant growth (+ 24%) and health (Kowalska et al., 2012). The effect was reverberated on the control of *Botrytis cinerea* also on stored fruits, extending their shelf-life without symptoms of damage up to 7 days. *Trichoderma* spp. isolates significantly reduced the infection of germinating seeds and carrots seedlings by *Pythium* spp. and efficiently influenced the growth of the seedlings as compared to the standard chemical seed dressing (Sobolewski et al., 2013). Similarly, foliar application of *T. asperellum* increased seed yield and weight and improved lipid content of organic oilseed rape (*Brassica napus* L.) (Kowalska, 2014). The mechanism of these plant growth promoting effects could be explained by the growth stimulation, observed with a *T. viridae* strain, particularly of lateral roots and inhibition of the elongation of hypocotyls, resulting in about fourfold increase of dry biomass in comparison to the control (Znajewska et al., 2018).

Among the bacteria exploited for protection against pathogens, the *Bacillus* and *Pseudomonas* genera have common commercial use and frequently are exploited also for plant growth promotion (Santoyo et al., 2012). A rich literature exists on these microorganisms (e.g., Kumar et al., 2011; Chowdhury et al., 2015; Islam et al., 2019) thus the reader is advised to refer to it. However, it is interesting to note that the entomopathogenic *B. thuringiensis*, was able to *in vitro* solubilize low-soluble inorganic phosphate and simultaneously produce IAA when formulated in k-carrageenan (Vassilev et al., 2006). This formulation boosted plant growth and P-uptake when introduced into a soil–plant system, stimulating the establishment and development of the co-inoculated endomycorrhizal fungus *Glomus deserticola* (Vassilev and Vassileva, 2004).

## Biocontrol Potential of Biofertilizers

Many plant growth promoters used for inoculation in cropping systems might serve as biocontrol microorganisms (Chowdhury et al., 2015; French, 2017; Table 1). The biocontrol potential of several P-solubilizers has been verified in several works of Vassilev and co-workers (Vassilev et al., 2006). Inoculation with *G. intraradices* significantly reduced the impact of the soil-borne pathogen *F. oxysporum* on tomato plants, paralleled by a significant decrease in the number of colony-forming units compared with the control treatment (Vassilev et al., 2008, 2009a). However, the further introduction of a filamentous fungus (*A. niger*) in the formulation, with different plant wastes and rock phosphate as microbial growing substrate, was more effective to control the pathogen. Similar results were achieved with *Phanerochaete chrysosporium* (Vassilev et al., 2009b) or in other field trials with P-solubilizing filamentous fungi against Fusarium wilt in tomato (Khan and Khan, 2001). In these cases, the biocontrol function was suggested to be based on production of hydrolytic enzymes or the competition for nutrients and space by the microbial P-solubilizers, as well as by hormones such as indole-3-acetic acid (IAA) and siderophores, being among the metabolites most frequently released by P-solubilizing microorganisms.

The observation that root colonization by AMF is not always associated to improved nutrition and increased vegetative biomass (Smith and Smith, 2011), has prompted to propose that improved stress tolerance is another major benefit of the symbiosis (Gianinazzi et al., 2010) and AM fungi are accepted as functioning in biocontrol (Johansson et al., 2004). Enhanced resistance of mycorrhizal plants to soil-borne pathogen attacks has been associated to the accumulation of phytoalexins, flavonoids, and isoflavonoids in AM-colonized root tissues (Ziedan et al., 2011; Jung et al., 2012). Interestingly, the bioprotective effect of *Glomus mosseae* against the soilborne pathogen *Fusarium oxysporum* f.sp. lycopersici was observed in different cultivars and genotypes which differed in their susceptibility to both the AMF and the pathogen (Steinkellner et al., 2012). Nevertheless, the response of phylogenetically diverse plants (i.e., tomato, soybean, and maize) to two mycorrhizal fungi – *Funneliformis mosseae* and *Rhizophagus irregularis* – depended on both the plant and the AMF species involved (Fernández et al., 2014). Although fungal pathogens reduce root colonization by AMF, the latter were shown to provide protection through increased enzymes activity, including those directly involved in the regulation of the symbioses. The biological protection of AMF has been also proved on plant parasitic nematodes, under greenhouse conditions: the population *of Meloidogyne incognita* or *Pratilenchus penetrans* was lowered by 45 and 87%, respectively, in mycorrhized roots in comparison to non-mycorrhized roots (Vos et al., 2012).

The effect of interactions between AMF and other agronomical practices shows how external factors can contribute to the expression of biocontrol potential. The interaction between AMF and different level of P availability was observed in the occurrence of *Alternaria solani* symptoms (Fritz et al., 2006). Mycorrhized tomato plants had significantly less *A. solani* symptoms than non-mycorrhizal plants, but increased P supply, which was paralleled to a reduction in mycorrhiza formation, led to a higher disease severity in mycorrhizal plants. On the other hand, individual co-inoculation of four *Glomus* species with *T. harzianum* affected the colony−forming capacity of the latter, but the combined inoculation – particularly with *G. intraradices* – resulted in a general synergistic effect on disease control (Martínez-Medina et al., 2009). The inoculation of bean plants with *Glomus mosseae*, besides decreasing propagule number *of Fusarium solani* in the rhizosphere, decreased pathogenic root rot by 34–77% (Dar et al., 1997). However, when co-inoculated with *Rhizobium leguminosarum*, the mycorrhized plants were more tolerant of the fungal root pathogen.

The induction of defense activity by AMF has been also proved in above ground tissues. *Helicoverpa arimigera* larvae feeding on leaves of tomato mycorrhized plants had a reduction of 62.3% in weight relative to non-inoculated plants, likely as a result of a priming effect related to jasmonate pathway (Song et al., 2013). Nevertheless, it could be speculated that the effect on above-ground herbivores derives also from reduced levels of metabolites connected to central catabolic and amino acid metabolism, particularly prominent in sink leaves, which prompted to suggest deteriorations rather than improvements in the nutritional value of colonized plants for higher trophic levels (Fester et al., 2011).

Several genera and species of bacteria (e.g., *Pseudomonas* or *Bacillus*) and fungi (e.g., Pennicillium or Aspergillus) ubiquitous in different soils are known to assist plants growth by mobilization of insoluble forms of K (Meena et al., 2014), with mechanisms similar to those found in P-solubilizers (Sheng and He, 2006). It is thus not unexpected that a strain of *Paenibacillus kribbensis* having potassium and phosphate-solubilizing capacity was also found to reduce the development of several cotton and wheat soil-borne pathogens *in vitro* (Zhang et al., 2013).

The potential function of plant-growth-promoting rhizobacteria in biocontrol has been long known and can be traced to bacterization studies with fluorescent pseudomonads beginning in the 1970s (Weller, 2007). Since then, a huge amount of studies has allowed to characterize the process of root colonization and the biotic and abiotic factors that are affecting it as well as the identification of genes and traits in bacterial fitness underlying the mechanisms of pathogen suppression (e.g., Labuschagne et al., 2010; Sayyed et al., 2013; Islam et al., 2019). However, notwithstanding this knowledge, the major difficulties and weakness in a broad use of PGPR strains in agricultural practices reside in formulation and registration of the bacteria for commercial use (Malusá et al., 2012; Bashan et al., 2014; Borriss, 2020).

## Regulatory Future Perspectives of Multifunctional Bioproducts

A sustainable agriculture is a central pillar of the United Nations Sustainable Development Goals (United Nations, 2015), which can be pursued by the wide adoption of microbial-based products in agronomical practices. The regulatory and policy pressure posed by this international agreement could potentially transform the market of bioproducts into a key segment of the world economy. Such potential is confirmed by recent market analysis reports, which valued at about 10.2 billion USD by 2025 the global biopesticide market, with an annual growth rate of about 15% (Anonymous, 2019b) and projected 3.15 billion USD by the end of 2026 for the biofertilizers market, at an annual growth rate of about 11% (Anonymous, 2019a). However, it is intriguing that for biopesticides, the market value projected for 2025 was already expected to be reached by 2017 (Marrone, 2007).

Most microbial-based PPPs present on the market have been designed for annual crops (mainly legumes and cereals), but there is an increasing demand for these products in fruit and vegetable crops, particularly for organic production. On the other hand, even though biofertilizers would not fully replace mineral fertilizers (Adesemoye et al., 2009), their application, possibly in association with organic fertilizers or other carbon-based products (Saeid and Chojnacka, 2019), could substitute to a large extent mineral or synthetic inputs, having also a positive impact on plant protection strategies.

However, the legal framework regulating the production and marketing of bioproducts can pose a bottleneck to their wider adoption because it reflects the incomplete knowledge on microbial-based products as well as precautions in their safety assessment. Considering the current situation in the EU, known to have a well-developed regulatory framework on agricultural inputs, it emerges that the biopesticide registration process and data requirements are similar to those needed for chemical pesticides (Regulation Eu 1107/2019). Even though a legal provision (Parliament, 2009) has introduced in the EU a compulsory integrated pest management since 2014 for all crops, the unfamiliarity with biologically based pest management of the risk assessors and regulators has not fostered the modification of the authorization process, taking into consideration the peculiarities of the biopesticides mechanisms of action, as it had already been suggested by prominent researchers (Chandler et al., 2011). However, recently, a specific working group has been organized to this aim, and also the European Food Safety Agency has actively operated to find new assessment methods (Council of the European Union, 2019). Interestingly, similar bottlenecks have been hampering biopesticides’ development and use also in the Indian context, paralleled with a legislation aimed to support bioproducts for organic farming which resulted in an unfair competition from sub-standard or misbranded biopesticides (Keswani et al., 2019a).

In case of biofertilizers, the rules have been enacted patchily in the world (Malusá and Vassilev, 2014) and in the European Union only in 2019 a provision has been enacted, though limiting the marketing to just four kinds of biofertilizers: three related to N nutrition (based on symbiotic Rhizobium spp. and free-living *Azotobacter* spp. and *Azospirillum* spp.) and one for P nutrition (based on mycorrhizal fungi) (Regulation Eu 1107/2019, 2019). The limitation of species allowed to be marketed as biofertilizers contrasts with the plethora of genera and species recognized having positive effects on plant nutrition and available for commercial applications (Umesha et al., 2018). Furthermore, the EU Regulation allows only the drying or freeze-drying processes in the formulation of the product, which is also restrictive considering the technological possibilities in this respect (Bashan et al., 2014; Vassilev et al., 2020).

Multifunctional bioproducts would also share with biopesticides and biofertilizers the issue of biosafety with respect to humans and the environment as, although only wild-type strains are being used for bioinocula development, the risk of pathogen spread cannot be completely excluded, thus requiring certain precautions (Keswani et al., 2019b).

In view of this situation, it appears quite difficult to expect that multifunctional bioproducts could soon be made available nor that manufacturers would advertise – not being it allowed by the legal provisions – either biocontrol or growth promotion features in bioproducts not registered for their respective purposes, even if the strains used could express them. The unlikeliness of a regulatory framework would also hamper the development of products based on microbial consortia that exhibit complementary and synergistic effects, through re-assembling strains with differing modes of action into small communities, thereby providing more consistent protection or growth promotion than with the application of single strains, which are now starting to gain attention as a possible strategy to widen the application of bioproducts (Reddy and Saravanan, 2013; Vassilev et al., 2015; De Vrieze et al., 2018). At the same time, the potential use of bioproducts for alleviating other abiotic stresses (Hassen et al., 2016; Rajendra Prasad et al., 2016), particularly relevant in the world-wide experienced climate change conditions, would also face difficulties due to lack of clear rules for their registration and marketing. The current regulatory framework in EU as well as that of other countries where bioproducts are highly promoted (see several articles in Singh et al., 2016) could be perceived as frustrating the researchers efforts in finding the best solutions to exploit microbial inocula, considering that plants (and animals) are no longer viewed as autonomous entities, but rather as “holobionts” (Bordenstein and Theis, 2015). Nevertheless, we believe that the research activity that is currently endeavored to better understand the biochemical and molecular mechanisms involved in plant–microbe–soil interactions, paralleled with their impact on the plant metabolomics and the interactions with endophytes, should also support the progress in manufacturing and the regulatory development, leading to the design and use of safe bioproducts with greater efficacy in enhancing the productivity of sustainable crops. To this aim, exploitation of endophytes (Fadiji and Babalola, 2020), or of pre-, pro-, and post-biotic approaches (Vassileva et al., 2020) as well as of the plants’ capacity to “Cry for Help,” i.e., recruit and subsequent assembly of protective specific microbiota (Bakker et al., 2018; Rodriguez and Durán, 2020), could represent possible research avenues to be explored.

## Author Contributions

JK, JT, and EM designed and drafted the work. KM and BT contributed to the revision of the manuscript. All authors contributed to the article and approved the submitted version.

## Conflict of Interest

The authors declare that the research was conducted in the absence of any commercial or financial relationships that could be construed as a potential conflict of interest.
